# Community-based survey on helminth infections in Kwilu province, the Democratic Republic of the Congo, and implications for local control strategies

**DOI:** 10.1371/journal.pntd.0008745

**Published:** 2020-10-28

**Authors:** Raquel Inocencio da Luz, Sylvie Linsuke, Clémentine Roucher, Alain Mpanya, Jane Nyandele, Nono Mubwa Mungwele, Bienvenue Nsiembele Mboma, Katja Polman, Epco Hasker, Marleen Boelaert

**Affiliations:** 1 Department of Public Health, Institute of Tropical Medicine, Antwerp, Belgium; 2 Epidemiology Unit, Institut National de Recherche Biomédicale, Kinshasa, Democratic Republic of the Congo; 3 Department of Biomedical Sciences, Institute of Tropical Medicine, Antwerp, Belgium; 4 Ministry of Health, PNLTHA, Kinshasa, Democratic Republic of the Congo; 5 Hubert Kairuki Memorial University, Dar es Salaam, Tanzania; 6 Ministry of Health, Kwilu, Democratic Republic of the Congo; Emory University, UNITED STATES

## Abstract

To adequately plan mass drug administration campaigns, the Democratic Republic of the Congo (DRC) needs further support for the mapping and monitoring of schistosomiasis (SCH) and soil-transmitted helminths (STH). We conducted a community-based survey in the health districts of Mosango and Yasa Bonga of the Kwilu province, DRC. A stratified two-stage cluster random sampling method was used to include participants into three different strata: Preschool-aged children (PSAC), school-aged children (SAC), and adults who were further subdivided into women of reproductive age (WRA) and other adults. In total, surveyors visited 30 villages, and 1 206 individuals participated in the study. Stool samples were collected to perform duplicate Kato-Katz smears for the detection of SCH and STH infection. Hookworm was the most prevalent infection in both districts, 34.1% (95%CI: 32.0–38.4), followed by *A*. *lumbricoides* (2.7%; 95%CI: 1.3–2.9) and *T*. *trichiura* (1.9%; 95%CI: 1.1–2.7). We did not find any SCH infection. The prevalence of each STH infection was similar across all risk groups, and the majority of the infected individuals was carrying light intensity infection. Compared to SAC, other adults were equally infected with hookworm. The prevalence of STH infection in SAC guides the MDA implementation because schoolchildren are most at risk and easily accessible program targets if school attendance is high. The current treatment strategy targets PSAC, SAC and WRA. However, this study shows that adults in general could also benefit from deworming. Therefore, community-wide preventive chemotherapy would be the most appropriate choice to control the hookworm burden rapidly.

## Author summary

Helminths are a group of intestinal worms that cause abdominal discomfort, diarrhea, and anemia due to blood loss in the stool. Regular mass drug administration (MDA) is one strategy to fight these worm infections. The appropriate MDA treatment scheme is chosen based on a population survey estimating the burden of infection. This survey is usually done in schoolchildren because they suffer the most from these infections and they are easy to reach through school infrastructures. However, one particular worm, the hookworm, is also highly present and clinically relevant in adults. We conducted a community-based survey in two districts of the Kwilu Province of the DRC. We found that hookworm was the predominant infection in the area and that adults were as often infected as the schoolchildren. Therefore, to effectively reduce hookworm infection, we advise extending treatment schemes to the entire community.

## Introduction

Soil-transmitted helminthiasis (STH) and schistosomiasis (SCH) affect more than 1 billion people, with the highest burden in the poorest regions of the world. STH are a group of Neglected Tropical Diseases (NTDs) that include hookworm (*Necator americanus* and *Ancylostoma duodenale*), roundworm (*Ascaris lumbricoides*) and whipworm (*Trichuris trichiura*) infections. Helminth eggs or larvae are transmitted through fecal contamination of soil. Humans are infected by the orofecal route, or by active skin penetration in the case of hookworm [1]. Infections lead principally to impaired nutritional status, diarrhea, abdominal discomfort, growth retardation, and reduced school and work performance [1–3]. SCH is caused by 6 species of trematodes: *Schistosoma guineensis*, *S*. *haematobium*, *S*. *intercalatum*, *S*. *japonicum*, *S*. *mansoni* and *S*. *mekongi*. The predominant causes of disease are *S*. *haematobium* and *S*. *mansoni*, widely prevalent in Africa [3]. Intestinal SCH caused by *S*. *mansoni* occurs after contact with fresh water contaminated with human excreta containing parasite eggs. A snail host must be present in the water to allow the parasite to complete its life cycle. The disease manifests as diarrhea and blood in the stool. In the advanced stage of the disease, enlargement of the liver and the spleen is seen [3].

The World Health Organization (WHO) produced a roadmap for control and elimination of SCH and STH based on a policy of preventive chemotherapy (PC) via mass drug administration (MDA) to decrease the worm burden at the population level [4]. For SCH, the goal is to treat at least 75% of school aged children (SAC) in schistosomiasis-endemic countries to reduce morbidity associated with SCH and eliminate SCH as a public health problem by 2020 [4–6]. For STH, the current guidelines focus on three high-risk groups to reduce morbidity, namely Preschool-aged children (PSAC), SAC, and women of reproductive age (WRA). The goal is to achieve by 2020 a minimum coverage of 75% of the most affected groups, PSAC and SAC, and to eliminate morbidity caused by moderate and heavy intensity STH infection (defined as the number of helminth eggs excreted by an individual exceeding a preset, species-specific threshold, used as a proxy for worm burden) to less than 1% [2, 7–9].The current guidelines recommend treating SAC, PSAC and WRA annually wherever STH prevalence ranges between 20% and 50% and twice a year where it exceeds 50% [7]. Combining PC strategies with water, sanitation, and hygiene (WASH) interventions is believed to contribute further to reduce helminth transmission. However, delivery and uptake by the targeted population is challenging and the impact of WASH is difficult to measure, resulting in heterogenic outcomes and evidence demonstrate mixed findings [10–12].

Despite the implementation of MDA, the disease burden remains high in many low- and middle- income countries [13]. Schools are considered as the best target for MDA programs because they provide an existing infrastructure to reach the age group for whom infection is often most intense and who might benefit the most from deworming at a stage when they are still learning and growing [14]. Although the current guidelines may be useful for reducing the morbidity of both *A*. *lumbricoides* and *T*. *trichiura*, they may not be as effective against hookworm [15]. It is well established that hookworm infections frequently predominate in adult populations [16, 17]. Moreover, the WHO has recently identified WRA as an additional risk group for hookworm infection [16], because it contributes to iron deficiency anemia. Men and elderly, not included in the current WHO guidelines, together with the ‘missed’ schoolchildren who do not attend school, are a potential source of reinfection for all age groups. As the post-2020 agenda for NTDs is under development, there is a growing interest in improving the morbidity control strategy, and when appropriate, shifting towards a more ambitious goal to interrupt transmission of STH [12, 17].

The Democratic Republic of the Congo (DRC) is a vast nation harboring a high prevalence of several major NTDs [18]. Despite recent efforts to provide reliable data on SCH and STH, data remain scarce as the exact burden remains unknown for several districts. Recent surveys indicate that both SCH and STH are still endemic in DRC, and highly endemic foci have been described [19–25]. Therefore, the DRC still needs further support for the mapping and monitoring of both diseases to plan MDA campaigns more adequately. Previous MDA campaigns from 2016 to 2018 have focused on PSAC and SAC with nationwide with coverage increasing from 76,9% to 91,8% in PSAC and from 61,3% to 77,51% in SAC [26]. Despite encouraging results, certain gaps still remain.

During a previous survey by our team in Mosango and Yasa Bonga in 2016, we noticed that SCH and STH were both prevalent in schoolchildren and that hookworm was the predominant infection exceeding 50% in both school populations [27]. With such a high burden in SAC, the environment is likely to be highly contaminated, putting the entire community at risk. As the prevalence of hookworm infection usually peaks in adulthood, we conducted another survey in the same Health Districts—at community level this time—to determine the prevalence, intensity and distribution of infection by age group of *S*. *mansoni*, *A*. *lumbricoides*, *T*. *trichiura* and hookworm. In between the studies, no MDA activities were implemented in this study area to treat SCH or STH, the district of Yasa Bonga benefits from ivermectin distribution to control Onchocerciasis.

## Methods

### Ethics

This study received ethical clearance from the Ethics Committee of the University Hospital of Antwerp, Belgium (B300201733778), and the Ethics Committee of the School of Public Health of Kinshasa (DRC ESP/CE/124/17). The two District Medical Officers (*Médecin-Chef de Zone*) were informed about the objectives, procedures, and potential risks and benefits of the study and gave their approval for the study.

We obtained written informed consent from the participants, and for minors, we obtained written informed consent from their parents or legal tutors. When participants, parents/tutors were unable to sign, we asked for a fingerprint as a token of consent. In addition to the parent’s consent, we also obtained assents for children 12–14 years of age. Community workers ensured that the participants were duly informed about the objectives, procedures, potential risks and benefits, and the concept of voluntary participation. We provided a single dose of anthelminthic treatment (albendazole, 400mg) free of charge when an STH infection was detected, according to WHO guidelines. When a SCH was detected, a single oral dose of praziquantel (40mg/kg) was provided free of charge.

### Study area, population and study procedure and analysis

**Fig 1:**
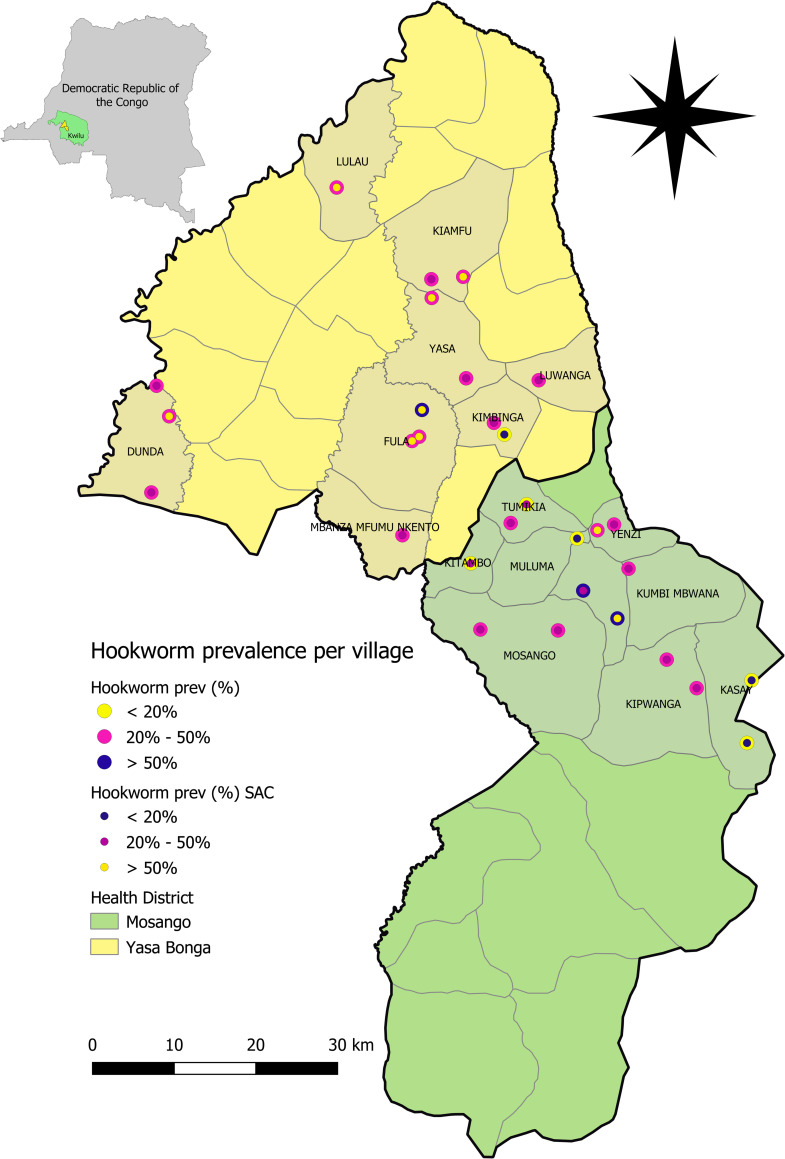
Map of the study area: The health districts Mosango and Yasa Bonga in the Kwilu province, DRC. Hookworm prevalence of surveyed individuals per village is shown and compared to the hookworm prevalence in SAC. (map generated using QGIS 2.18.2).

We conducted a random household survey from 29^th^ of November until the 14^th^ of December 2017 in the two Health Districts (Mosango and Yasa Bonga), situated in the province of Kwilu, DRC (Fig 1). The province of Kwilu is located to the east of the capital Kinshasa. Apart from the capital Kikwit and the town of Bandundu, the province is essentially rural. The climate is tropical with two seasons, a rainy season from October until May and a dry season from June until September. The Kwilu province is divided into 19 Health Districts which are further divided into Health Areas. The Mosango health district has a surface of 3 350km^2^, has an estimated population of 111,128 inhabitants and is subdivided into 16 Health Areas. Yasa Bonga has a surface of 2 810 km^2^ with an estimated population of 180,439 and 20 Health Areas [28]

A stratified two-stage cluster random sampling method, adapted from the sampling procedure developed by the Expanded Program on Immunization (EPI) method [29], was used. The village was the primary sampling unit and individuals were selected within three strata: 1) PSAC (1–5 years); 2) SAC (6–14 years) and 3) adults (≥18 years). WRA, defined as women 15–49 years old, were not separately sampled as a stratum but considered as a group during data analysis, adults were thus subdivided into WRA and ‘other adults’, therefore WRA in our sample are 18–49 years old.

The sample size calculation was done using Epi Info 7.2 and based on the expected prevalence of hookworm of 50% as observed in a previous survey in the same area [27]. Defining the expected precision at 7.5%, with a confidence interval of 95% and a design effect of 2 [29], the resulting minimum sample size per stratum was 360. A margin of 10% (n = 36 individuals) was added to compensate for any absentees or refusals. As a result, the desired sample size was 396, or 30 clusters of 13 individuals. In each cluster, individuals were recruited for the three strata, resulting in a total minimum sample size of 1 170 individuals.

Thirty villages were randomly selected within the two districts (15 per district) using an exhaustive village list in Excel and the command “Rand()”. This command generates a random number and for each village in the list we generated a random number. The villages were then sorted by increasing order according to the generated number and the first 15 villages per district were selected. Upon visiting the village, the first household was selected by spinning a bottle at the village chief’s house and subsequent households were included following the same direction until cluster size was reached. Participants enrolled in each stratum were kindly requested to provide one stool sample for parasitological examination.

### Laboratory procedures

Stool samples were shipped to the laboratory of the General Reference Hospital of the District for parasitological examination. Duplicate Kato-Katz thick smears were prepared from each stool sample using 25mg templates [30]. The smears were allowed to clear for at least 30 min and a maximum of 1 hour before the first examination under the microscope for hookworm infection and egg-count. After that, the smears were examined 24h later to determine *S*. *mansoni*, *T*. *trichiura* and *A*. *lumbricoides* infection. The number of eggs was counted and recorded by experienced technicians. Eggs per gram of feces (EPG) was calculated by multiplying the number of counted eggs in the two slides by 20. WHO guidelines [31] were used to classify intensity of infection of *S*. *mansoni* as light (1–99 EPG), moderate (100–399 EPG) and heavy infection (≥ 400 EPG). For STH, the intensity of infection was also classified according to the WHO guidelines [31]: for. *A*. *lumbricoides* light (1–4 999 EPG), moderate (5 000–49 999 EPG) and heavy infection (≥ 50 000 EPG). For *T*. *trichiura* light (1–999 EPG), moderate (1 000–9 999 EPG) and heavy infection (≥ 10 000 EPG) and for hookworm light (1–1 999 EPG), moderate (2 000–3 999 EPG) and heavy infection (≥ 4 000 EPG). Finally, to calculate the prevalence of the combined STH infection as recommended by the WHO, the mathematical formula according to de Silva *et al*. was used [32].

### Data analysis

Statistical analysis was performed with STATA, version 14 (StataCorp. College Station, USA). Two-stage cluster survey analysis was performed using the village as primary sampling unit and specific sampling weights calculated for each strata of each village. Sampling weights where calculated taking into account the number of selected and total villages, number of participants selected and the total population of each stratum. The total population of each stratum was calculated based on the total population of each village and demographic age distribution as published in the demographic health survey of the DRC [25].

Descriptive statistics are presented using frequencies, proportions and 95% Confidence Intervals (95% CI). The mean ± standard deviation (SD) was computed for normally distributed data, else the median and interquartile range (IQR) is presented. Bivariate survey analysis was performed to investigate whether significant differences in prevalence could be found compared to SAC as the reference group. Differences were considered statistically significant if p < 0.05.

Finally, we calculated the prevalence of infection for each village and categorized them according to the WHO guidelines. Villages were defined as: low risk (<20%) needing no intervention, moderate risk (20–50%) requiring MDA once a year and high risk (>50%) or MDA twice a year [31]. For each village, we compared the prevalence of infection of all the surveyed individuals to the prevalence in the SAC.

## Results

### General characteristics of the study population

The survey team visited thirty villages in the two Health Zones. These were distributed across 9 Health Areas in Mosango and 8 Health Areas in Yasa Bonga (Fig 1). In total 1,211 individuals were enrolled in the study, of which 618 (51.2%) were female. Five individuals did not provide samples and were excluded from the analysis resulting in a final sample size of 1,206 participants, 616 in Mosango, and 595 in Yasa Bonga. However, microscopic results of hookworm infection were only available for 1,197 individuals, 612 in Mosango, and 585 in Yasa Bonga. The discrepancy was due to misplacement of the slides in the laboratory, 9 slides could not be read on time. The study population consisted of 390 (32.3%) PSAC, 418 (34.6%) SAC, 147 (12.2%) WRA and 251 (20.8%) other adults. Within each age category, the median age was four years (IQR:3–5) in PSAC, 8 (IQR:7–10) in SAC, 30 (IQR:24–38) in WRA, and 48 (IQR:32–59) in other adults.

### Prevalence of infection

Table 1 shows the prevalence of SCH and STH in the study population. None of the individuals presented a SCH infection. However, STH were prevalent in both districts and the survey-wise prevalence of STH was 35.0%. Hookworm was the most prevalent infection in both districts, 34.1% (95%CI: 32.0–38.4), followed by *A*. *lumbricoides* (2.7%; 95%CI: 1.3–2.9) and *T*. *trichiura* (1.9%; 95%CI: 1.1–2.7). The prevalence of STH was quite similar in both districts. The Kato Katz revealed *Enterobius vermicularis* infection even though this method is not intended for the detection of this species, eggs were detected in the fecal material. The prevalence was 0.6% (95%CI: 0.4–2.3) in both districts, 1.1% (95%CI: 0.04–2.3) in Mosango and 0.2% (95%CI: 0.0–0.9) in Yasa Bonga.

**Table 1:** The prevalence of SCH and STH infection in the study population.

	Overall (N = 1206)			Mosango (N = 616)			Yasa Bonga (N = 590)		
	n	%	95%CI	n	%	95%CI	n	%	95%CI
*S*. *mansoni*	0	-	-	0	-	-	0	-	-
*T*. *trichiura*	24	1.9	1.1–2.7	7	1.1	0.04–2.1	17	2.8	1.9–5.3
*A*. *lumbricoides*	32	2.7	1.3–2.9	4	0.7	0.2–1.9	28	4.8	3.0–6.6
Hookworm*	408	34.1	32.0–38.4	193	31.5	20.7–39.3	215	36.8	31.5–40.3
*E*. *vermicularis*	8	0.6	0.4–2.3	7	1.1	0.04–2.3	1	0.2	0.0–0.9

* N = 1197: 612 in Mosango & 585 in Yasa Bonga 95%CI: 95% Confidence Interval

The STH prevalence per village classified them all as low-risk areas for both *T*. *trichiura* and *A*. *lumbricoides* infection. The prevalence of hookworm infection was more diverse. Nearly all villages in the Yasa Bongo district were classified as moderate risk, while the district of Mosango showed a mix of the three categories (Fig 1). Interestingly, villages from the same health area were mostly classified in the same category. Both villages from the Mwanda Koso area were classified as high risk and both villages of Kasay area were classified as low risk. Next we compared further the classification based on the hookworm prevalence of all the individuals in the village compared to the classification based on the hookworm prevalence in SAC. The classification of the villages matched in 66.7% (20/30) of the cases. When discordance occurred, the village usually shifted from moderate risk overall to high risk when only SAC were taken into account (Fig 1)

**Fig 2:**
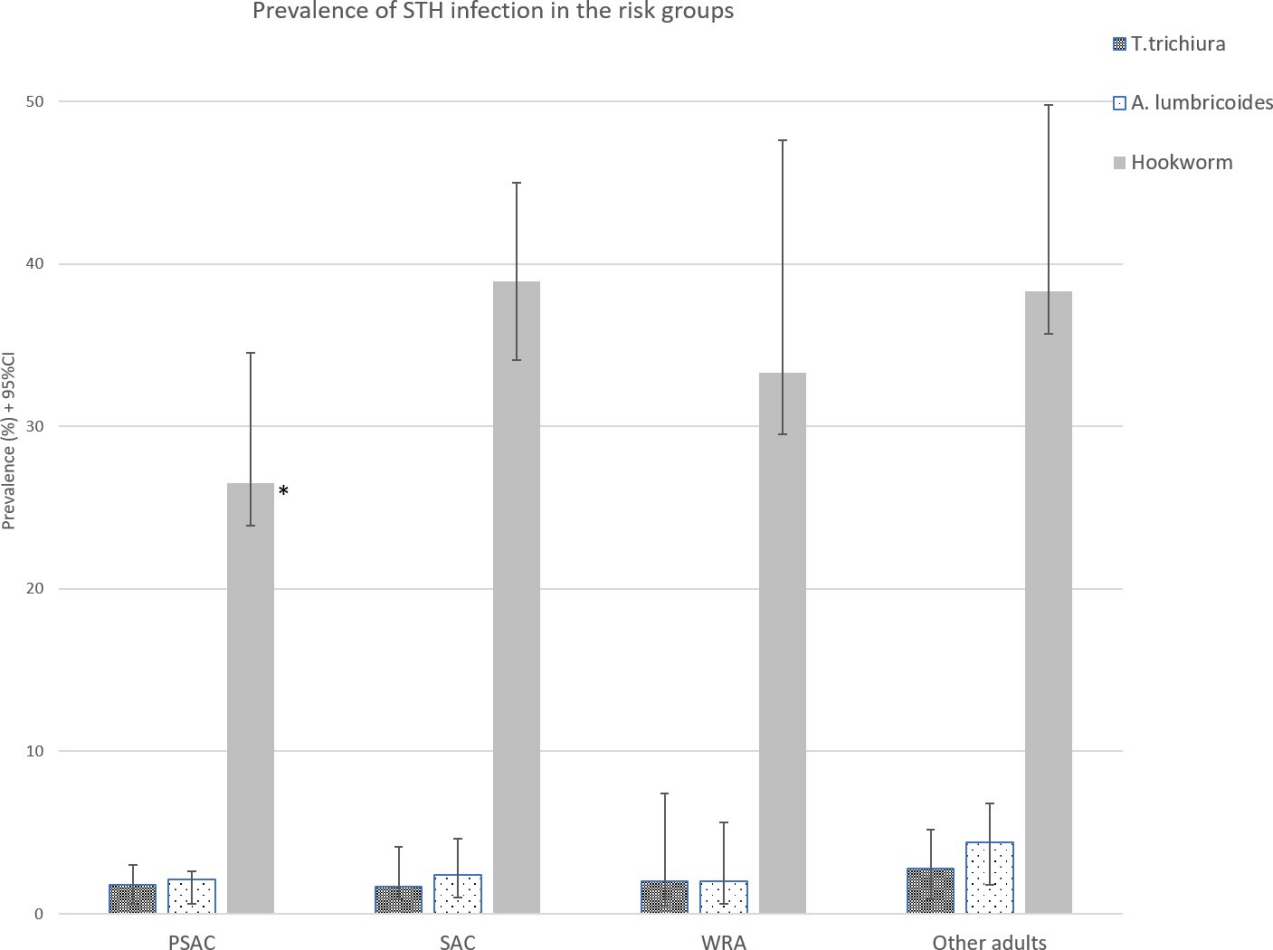
Prevalence of STH infection by risk groups PSAC: pre-school aged children, SAC: school aged children, WRA: women of reproductive age and other adults. * The prevalence of hookworm infection was significantly less in PSAC compared to SAC.

Fig 2 shows an overview of the prevalence of STH infections among the different risk groups in the study population. The prevalence of each STH infection was similar across the risk groups. For both *T*. *trichiura* and *A*. *lumbricoides* infection the highest prevalence, 2.8% (95%CI: 0.9–5.2) and 4.4% (95%CI: 1.8–6.8) respectively, was found in other adults. This pattern was consistent over both districts, with Yasa Bonga bearing a higher burden of *T*. *trichiura* and *A*. *lumbricoides* compared to Mosango (S1A Table). Hookworm infection was prevalent in all age categories with 26.5% (95%CI: 23.9–34.5) in PSAC, 38.9% (95%CI: 34.1–45.0) in SAC, 33.3% (95%CI: 29.5–47.6) in WRA and 38.3% (95%CI: 35.7–49.8) in other adults. In Mosango, the highest hookworm prevalence was found in other adults (40.3%; 95%CI: 38.8–57.8), while in Yasa Bonga it was found in the SAC (43.2%, 95%CI: 36.3–51.0). The prevalence of STH in SAC was not significantly different to the prevalence of STH in the other risk groups. There was one exception, the prevalence of hookworm was significantly less in PSAC (Fig 2; S1A Table).

### Infection intensity

**Fig 3:**
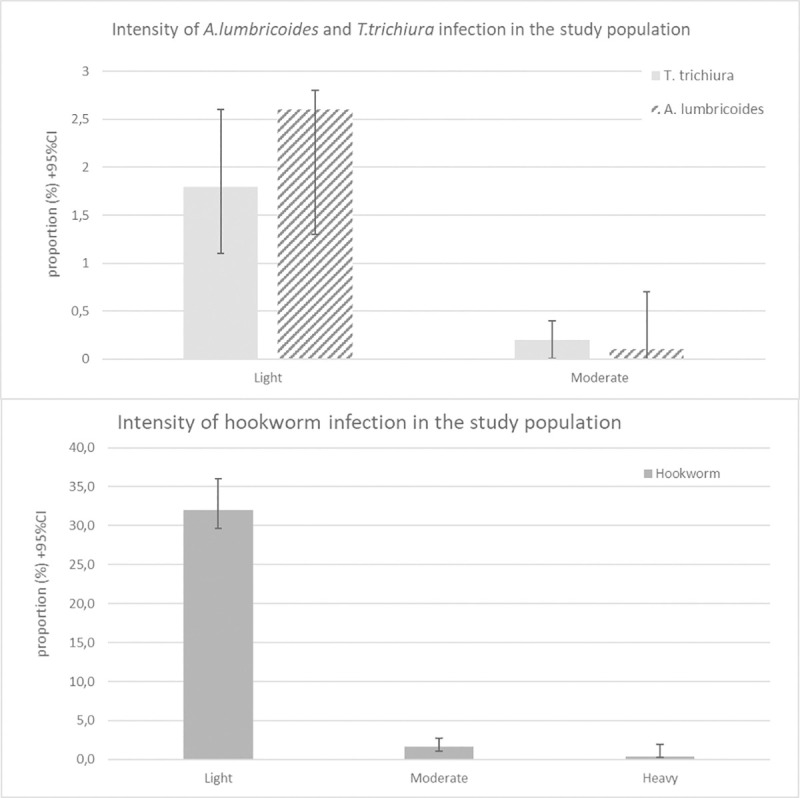
Intensity of STH infection in the study population. Classification of intensity of infection for A. lumbricoides light (1–4 999 EPG), moderate (5 000–49 999 EPG) and heavy infection (≥ 50 000 EPG). For T. trichiura light (1–999 EPG), moderate (1 000–9 999 EPG) and heavy infection (≥ 10 000 EPG) and for hookworm light (1–1 999 EPG), moderate (2 000–3 999 EPG) and heavy infection (≥ 4 000 EPG).

The majority of the infected individuals were carrying light STH infections (Fig 3), this was similar across the risk groups and across the districts (S2B Table). Moderate infection rates of *T*. *trichiura* and *A*. *lumbricoides* were below 1% across the risk groups and in both districts. Among the hookworm infected individuals, the majority was carrying light infections and moderate infection rates ranged between 0.8% (95%CI: 0.2–2.3) and 2.4% (95%CI: 1.3–5.4). Heavy hookworm infection was only seen in 5 individuals, all originating from the Mosango district (S2B Table).

### Co-infections

In the study population 24 individuals (2.0% 95%CI: 1.0–2.4) were infected with two parasites and three individuals (0.3%; 95%CI: 0.1–0.7) were carrying all three STH species. The most common co-infection was hookworm in combination with either *T*. *trichiura* or *A*. *lumbricoides*.

## Discussion

This survey, conducted at the community level in two Health Districts of the Kwilu province, showed that STH infection was prevalent in the study population, hookworm was particularly highly prevalent. No *S*. *mansoni* infection could be found, although, in the previous survey in the same area, a prevalence of 8.9% was found in SAC [27]. SCH was thus not a public health problem in the area and transmission might be somewhat limited. Other possible explanations could be by schistosomicidal effect of antimalarial drugs, improved access to clean water or combination of both. The DRC is highly endemic for malaria [33], its first line treatment policy for uncomplicated malaria is Artesunate-Amodiaquine (AS-AQ) which has shown to reduce *S*. *mansoni* infection [34]. Both districts have benefited from restauration of clean water sources and WASH services were supplied in Mosango district in 2015–2017 by the NGO Memisa (personal communication: dr. Raphael Cikuya) that could have further emphasized reduction of SCH infection.

The prevalence of STH infections in SAC is used to guide the implementation and frequency of PC. This study area would, therefore, require annual MDA at the district level due to hookworm infection reaching 38.9% in SAC. However, the proportion of infected WRA and other adults in this survey were similar to the SAC (Fig 2). Recent adaptations to the guidelines now include PSAC, SAC and WRA as at-risk populations to target for MDA [7]. WRA have been included as a target for PC due to hookworm infection that is worsening iron deficiency anemia [16]. However, men and the elderly are currently not included in PC programs. We observed that compared to SAC, adults other than WRA were equally infected with hookworm (Fig 2). In combination with the fact that the age-specific hookworm prevalence increases with age [16], the impact of school-based deworming on overall community-wide prevalence seems limited [8, 35]. Several models have suggested expanding to community-wide MDA, especially if hookworm is the predominant infection, to diminish the probability of reinfection while reducing worm burden more efficiently [2, 36–38]. In addition, risk factors related to hookworm infection and morbidity in adulthood should be further investigated.

The implementation of PC programs in a vast country such as the DRC can be very challenging because the geographical area to cover is large and remote villages are hard to reach due to poor road infrastructure in rural areas. To deliver health services, each province is divided into health districts, under the management of the District Medical Officer, which are further subdivided into health areas. For the two surveyed districts, Mosango and Yasa Bonga, we mapped the hookworm infection rate per village and compared the hookworm prevalence found in all surveyed individuals to the hookworm prevalence within SAC (Fig 1). In the majority of the cases, the prevalence matched between the two groups and the current PC recommendation remain unchanged. In case of a discordance, it would be more beneficial for the village to base the PC recommendation on the prevalence in SAC because the infection rate in SAC was usually higher. The hookworm prevalence in SAC is thus a good proxy for the prevalence in the whole community. We also noticed that hookworm infection was quite homogeneously spread over the Yasa Bonga district, while the distribution was more heterogeneous in the Mosango district. However, villages belonging to the same health area were usually categorized in the same way. In this context, it might, therefore, be most appropriate to manage the implementation of MDA, monitoring and evaluation activities at health area level instead of the district level.

The intensity of infection is an important parameter to measure the impact of PC as the new WHO goal is to reduce moderate and heavy infection prevalence below 1% in all risk groups [7]. STH infections of low intensity have limited or non-specific symptoms, thereby generally considered asymptomatic [1, 13]. In this study population, this goal can be considered reached for *A*. *lumbricoides* and *T*. *trichiura* infection. Hookworm infection, on the other hand, exceeded the 1% threshold in SAC (2.4%), WRA (1.4%) and other adults (2.0%) for moderate intensity of infection although heavy infection was less than 1% (Fig 3). Even though moderate and heavy infection rates were low, individuals carrying light infection were numerous. These persons with chronic infections could still manifest functional symptoms and remain at continuing risk to accumulate worms over time, so even if the impact is low, they would still gain health benefits from PC [13].

We recognize that the current egg counting methods suffer from measurement error in samples with low infection intensity that can be missed [39–41]. However, single-sample is the method recommended by WHO and is universally used in the monitoring and evaluation of deworming programs [42]. It is the relation between morbidity and intensity of infection that remains quite unclear as light infections can be associated with non-negligible morbidity, and the severity of symptoms associated with moderate to high-intensity infection is highly variable [40]. Morbidity was, however, not measured during this study, and its correlation with the intensity of infection needs further investigation.

As the 2020 timeline to control morbidity is nearing its end, the interest is growing to move towards the 2030 target of disruption of the transmission of STH. Although remarkable progress has been made globally for MDA coverage and reduction of morbidity, some gaps remain in some regions of the world. In 2017 the DRC did not reach the 75% PC coverage for SAC, but this target has been reached in 2018 [3, 6]. Yet, it is still under debate that the current target is not even sufficient to control hookworm infection because hookworm infection usually predominates in the adult population [2, 8, 9, 15, 16, 43]. Indeed, our study confirms that adults were equally infected as the SAC, acting as reservoirs when excluded from the control program. Several mathematical and economical models have, in addition, shown that expanding MDA to all individuals results in greater reductions in STH prevalence. This applies even to children at the community level compared to targeted treatment of SAC and PSAC only [8, 9, 43]. Community-wide MDA could, in addition, have a larger impact on hookworm reservoirs during a shorter timeframe, while targeted deworming programs will need to continue indefinitely due to continued transmission at the community level [37, 43]. The immediate consequence of extending MDA coverage is a substantial financial investment for increased drug donation, drug delivery and improved epidemiological surveillance. Even though the cost will substantially increase, community-wide MDA is highly cost-effective because of its averted morbidity, even if transmission is not eliminated with the condition of broad inclusion and high coverage[12, 44, 45].

Many questions remain regarding how best to deliver STH treatment programs to achieve the greatest impact, including which age groups should be targeted and how often. It will also be important to consider how different program aims (such as morbidity control versus reduction in transmission) may require different effectiveness metrics, as this will impact the optimum strategy to use [17, 36]. In the context of Mosango and Yasa Bonga, hookworm was, the only species that required a public health intervention in the study area. General sanitary improvement such as WASH programs could additionally also play a crucial role for sustainable control and possible disruption of transmission[2]. Another challenge to overcome is the potential emergence of resistance to benzimidazoles, mainly due to the lack of availability of second-line treatment options [8]. The final challenge will be to prevent persistence of transmission and risk of resurgence to truly prevent disease [13].

## Conclusion

Several authors advocate for a revision of the PC guidelines when hookworm is the predominant infection [43]. Systematically excluding a segment of the community from treatment can potentially undermine control programs that seek reduction of morbidity in the short term and elimination in the longer term [14]. Even though WRA has recently been added as an additional group to target for PC, we demonstrated that non-WRA adults are equally infected as SAC and WRA, with the exception of PSAC. Community-wide preventive chemotherapy would be the most appropriate choice to rapidly control morbidity. On the long term it could lead to possible disruption of transmission, especially in combination with additional WASH interventions.

## Supporting information

S1 Table A: Prevalence of STH infection in the different risk group. (PDF)

S2 Table B: Intensity of infection by risk groups across the districts. (DOCX)
