# Supplementary material for: Community-based survey on helminth infections in Kwilu province, the Democratic Republic of the Congo, and implications for local control strategies
Source: PLoS Negl Trop Dis. 2020 Oct 28;14(10):e0008745. doi: 10.1371/journal.pntd.0008745 (PMC7592847; doi:10.1371/journal.pntd.0008745)
Supplement: S1 Table — (PDF) [file pntd.0008745.s001.pdf]

Supporting information

Both districts	PSAC (n=390)				SAC (n=418)			WRA (n=147)				other adults (n=251)			
	n	%	95%CI	p-value	n	%	95%CI	n	%	95%CI	p-value	n	%	95%CI	p-value
T. trichiura	7	1.8	0.6-3.0	0.5	7	1.7	0.9-4.1	3	2.0	0.5-7.4	0.9	7	2.8	0.9-5.2	0.8
A. lumbricoides	8	2.1	0.6-2.6	0.3	10	2.4	1.0-4.6	3	2.0	0.6-5.6	0.9	11	4.4	1.8-6.8	0.3
Hookworm*	102/385*	26.5	23.9-34.5	**0.009**	161/414*	38.9	34.1-45.0	49	33.3	29.5-47.6	0.8	96	38.3	35.7-49.8	0.5
Mosango	PSAC (n=196)				SAC (n=215)			WRA (n=81)				other adults (n=124)			
	n	%	95%CI	p-value	n	%	95%CI	n	%	95%CI	p-value	n	%	95%CI	p-value
T. trichiura	1	0.5	0.0-3.6	0.2	2	0.9	0.4-2.1	2	2.5	0.7-1.1	0.3	2	1.61	0.3-6.0	0.8
A. lumbricoides	1	0.5	0.0-1.0	0.9	1	0.5	0.1-7.1	0	0	-	-	2	1.6	0.2-6.0	0.3
Hookworm*	42/192*	21.9	20.7-35.3	0,06	75	34.9	30.0-45.1	26	32.1	28.9-52.3	0.6	50	40.3	38.8-57.8	0.08
Yasa Bonga	PSAC (n=194)				SAC (n=203)			WRA (n=66)				other adults (n=127)			
	n	%	95%CI	p-value	n	%	95%CI	n	%	95%CI	p-value	n	%	95%CI	p-value
T. trichiura	6	3.1	1.2-6.9	0.6	5	2.5	1.6-9.4	1	1.5	0.0-2.4	0.6	5	3.9	1.4-10.0	0.9
A. lumbricoides	7	3.6	1.6-7.2	0.6	9	4.4	2.1-8.7	3	4.6	1.8-16.7	0.7	9	7.1	3.8-14.7	0.3
Hookworm*	60/193*	31.1	24.7-39.5	**0.03**	86/199*	43.2	36.3-51.0	23	34.8	22.5-47.7	0.2	46	36.2	25.4-43.3	0.1

PSAC: Pre-school aged children, SAC: school aged children, WRA: women of reproductive age. Significant differences in prevalence, compared to the SAC, was investigated, p-values in bold indicate a significant difference.

S1 Table A: Prevalence of STH infection in the different risk group
