# Supplementary material for: Community-based survey on helminth infections in Kwilu province, the Democratic Republic of the Congo, and implications for local control strategies
Source: PLoS Negl Trop Dis. 2020 Oct 28;14(10):e0008745. doi: 10.1371/journal.pntd.0008745 (PMC7592847; doi:10.1371/journal.pntd.0008745)
Supplement: S2 Table — (DOCX) [file pntd.0008745.s002.docx]

*PSAC: Pre-school aged children, SAC: school aged children, WRA: women of reproductive age*

*Classification of intensity of infection for A. lumbricoides light (1 - 4 999 EPG), moderate (5 000 – 49 999 EPG) and heavy infection (≥ 50 000 EPG). For T. trichiura light (1-999 EPG), moderate (1 000 – 9 999 EPG) and heavy infection (≥ 10 000 EPG) and for hookworm light ( 1 – 1 999 EPG), moderate (2 000 – 3 999 EPG) and heavy infection (≥ 4 000 EPG).*

***S2 Table B:*** Intensity of infection by risk groups across the districts
